# Long-term tooth discoloration induced by apical plugs with hydraulic calcium silicate-based cements in bovine teeth with open apices – a 6-year in-vitro study

**DOI:** 10.1007/s00784-025-06407-0

**Published:** 2025-06-05

**Authors:** Ralf Krug, Fabian Schwarz, Britta Hahn, Marcel Reymus, Florin Eggmann, Gabriel Krastl, Sebastian Soliman

**Affiliations:** 1 Department of Conservative Dentistry and Periodontology and Center of Dental Traumatology, University Hospital of Würzburg, Pleicherwall 2, 97070 Würzburg, Germany; 2 Department of Conservative Dentistry and Periodontology, University Hospital, Ludwig-Maximilians-University, Munich, Germany; 3 Department of Periodontology, Endodontology and Cariology, University Center for Dental Medicine Basel UZB, University of Basel, Basel, Switzerland

**Keywords:** Apexification, Apical plug, Bovine teeth, Calcium-silicate based cement, Mineral trioxide aggregate, Open apex, Portland cement, Root end closure, Spectrophotometer

## Abstract

**Objectives:**

To investigate the long-term tooth discoloration induced by different hydraulic calcium silicate-based cements (HCSCs), depending on blood contamination and placement method in-vitro.

**Materials and methods:**

Eighty bovine teeth, sliced to a length of 18 mm (crown 8 mm, root 10 mm), were randomly assigned to 10 groups (*n* = 8), receiving ortho- or retrograde apical plug treatment (APT). Apical plugs were 4 mm in length and made of ProRoot MTA (Dentsply), Medcem MTA (Medcem), TotalFill BC RRM Fast Set Putty (Brasseler), or Medcem Medical Portland Cement (Medcem) plus bismuth oxide (Bi_2_O_3_) with and without bovine blood. Orthograde (with or without preoperative adhesive coronal dentin sealing) and retrograde APT were compared. Teeth were root-filled with gutta-percha and sealer, adhesively restored, and stored in distilled water. Tooth color was measured on apical plug, gutta-percha/sealer, and crown surface before treatment, after 24 h, and up to 72 months after treatment by spectrophotometry. Color difference (ΔE) values were calculated and analyzed by ANOVA with post-hoc-tests, Shapiro-Wilk-test, Friedman-test, Mann-Whitney-U-test, t-test, and post-hoc-tests with Bonferroni correction (α = 0.05).

**Results:**

The increase of tooth discoloration occurred in all groups with no significant differences between HCSCs (*p* >.05). After six years, color changes were strongly marked on roots but insignificant on crowns. The color differences on the measuring surface of the apical plug were statistically significant between 24 and 72 months (*p* <.001). Blood had a more relevant impact on tooth color than Bi_2_O_3_. There were no substantial long-term effects of retrograde placement (*p* >.05) or preoperative dentin sealing (*p* >.05).

**Conclusions:**

Apical plugs of the tested HCSCs cause ongoing discoloration of bovine roots, but no discoloration of bovine tooth crowns within a six-year period.

**Clinical relevance:**

Apical plugs should be carefully placed. If direct contact with the coronal dentin is avoided, long-term aesthetic impairments are unlikely to occur.

## Introduction

Single-visit apexification using hydraulic calcium silicate-based cements (HCSCs) is an effective endodontic treatment approach for managing teeth with wide open apices requiring root canal treatment. Mineral trioxide aggregate (MTA), a type of HCSC with a well-established clinical performance record, has been used for root-end closure for over three decades. This method offers the advantage of completing both endodontic and restorative treatment more rapidly compared with conventional apexification procedures [1]. In addition to offering a more straightforward treatment approach, root-end closure with so-called MTA plugs reduces the risk of tooth fractures. Clinical studies have reported success rates exceeding 90% within five years of endodontic therapy using MTA plugs placed near open apices [2–8].

However, despite the well-documented advantages of HCSCs for single-visit apexification, these materials present several clinically significant limitations, such as challenging handling characteristics and the potential for tooth discoloration [1]. To mitigate the risk of tooth discoloration, it is essential to carefully place HCSC materials using appropriate application tools that facilitate precise and targeted delivery [9–11].

Laboratory studies have shown that HCSCs can interact with blood or sodium hypochlorite [12–16]. It has been suggested that white MTA and bismuth oxide (Bi_2_O_3_) radiopacifiers contribute to tooth discoloration upon contact with sodium hypochlorite [13, 16, 17]. Additionally, the discoloration potential of HCSCs is heightened if the setting process occurs in the presence of blood [14, 15, 18, 19].

Owing to their potential for tooth discoloration, the placement of HCSC apical plugs can lead to aesthetic impairments [20, 21]. Notably, attempts to mitigate HCSC-related discoloration by sealing pulp chamber walls with a dental adhesive were ineffective in a bovine tooth model [22]. This finding suggests that contact between HCSC materials and coronal dentin should always be avoided. Therefore, the placement of these materials must be carefully executed near the apical foramen under clinical conditions to prevent aesthetic compromise.

A laboratory study investigating discoloration following HCSC placement within bovine root canals reported a noticeable increase in HCSC-related discoloration on the root, with a lesser degree observed in the coronal aspect of the tooth within the two-year observation period [22]. However, there is a paucity of long-term data regarding coronal discoloration after HCSC placement with the root canal. Consequently, it remains uncertain whether these apical plug materials affect the color of the tooth crown and thus compromise dental aesthetics over an extended period.

To address this knowledge gap, the present study extends the observation period to six years, building on previous research. The aim of this study is to assess the long-term tooth discoloration potential of four HCSC materials. In addition to comparing these materials, the study specifically aims to examine the effects of factors such as blood contamination, presence of Bi_2_O_3_, dentin sealing prior to HCSC placement, and the technique of HCSC placement (orthograde versus retrograde). Long-term in-vitro studies are able to investigate adverse effects of poor long-term stability and exsolution of HCSCs followed by the diffusion and subsequent chemical reactions with the components of the root canal filling within the interface to the dentine. One research question was stated: do the four tested HCSCs induce aesthetically significant tooth discoloration over the long term?

## Materials and methods

### Specimen preparation

This experimental study was conducted in conformity with the principles set forth in the WMA Statement on Animal Use in Biomedical Research. The study setup was previously described by Krug et al. in detail [22]. Eighty bovine incisors from cattle 4 to 6 years of age were extracted and stored in a 1% chloramine-T solution at room temperature. The periodontal ligament of each tooth was removed with scalpels and gauze pads. Teeth were then sectioned to a standard crown length of 8 mm and a root length of 10 mm using a diamond saw. Calipers (Karl Hammacher, Solingen, Germany) were used to measure the distances between each cut surface and the most apical point of the cementoenamel junction (CEJ) on the labial side of each tooth.

Pulp tissue was manually removed using an ISO 60 stainless steel file (Dentsply Maillefer, Ballaigues, Switzerland). The root canals were subsequently rinsed with distilled water. The root canals were then reamed to a standard diameter of 2.3 mm using a bur (410 RFX 023, Busch, Engelskirchen, Germany). To create a flat surface for the measuring probe of the digital spectrophotometer to rest on, a cylindrical diamond bur (No. 030301, 6 mm diameter, Busch, Engelskirchen, Germany) was used to flatten the outer vestibular convexity. Each specimen was then stored in distilled water.

Following root canal preparation, endodontic irrigation was performed in an ultrasonic bath: 24 h before root canal obturation, the specimens were immersed in 3% sodium hypochlorite for 30 min, followed by 20% ethylenediaminetetraacetic acid for 2 min, and then 3% sodium hypochlorite for 3 min. The root canals were rinsed with distilled water between each irrigation step. It was done manually a random allocation of the samples for the following tested materials.

Four commercial HCSC products were investigated as reported previously [22]:

1. ProRoot MTA (Dentsply Tulsa Dental, Tulsa, USA), the first commercial HCSC product used to perform root-end closure in teeth with wide apices [1, 23, 24], contains 75% Portland cement, 5% calcium sulfate dehydrate, and 20% Bi_2_O_3_ as the radiopacifer [25].
2. Medcem MTA (Medcem, Weinfelden, Switzerland), a so-called second generation MTA, consists of Pure Portland Cement with zirconium oxide as the radiopacifer. It is recommended for perforation repair [26], apexification [27], surgical endodontics [28], pulp capping [29], and pulpotomy [30]. Medcem MTA root-end-fillings have shown low leakage [31].
3. Medcem Medical Portland Cement (MMPC) (Medcem, Weinfelden, Switzerland) is recommended for direct and indirect capping of permanent and primary teeth and for pulpotomy, perforation repair and apexification [32]. It contains only Portland cement without additional ingredients.
4. TotalFill BC RRM Fast Set Putty (Brasseler, Lemgo, Germany), a high-viscosity modification of the TotalFill BC Sealer, contains only calcium sulfate and tantalum pentoxide instead of calcium hydroxide.

The specimens were randomly allocated to ten groups, each consisting of eight specimens. All teeth were filled apically with one of four tested HCSCs with or without the admixture of bovine blood alternately in random order (Table 1) [22].

**Table 1:**
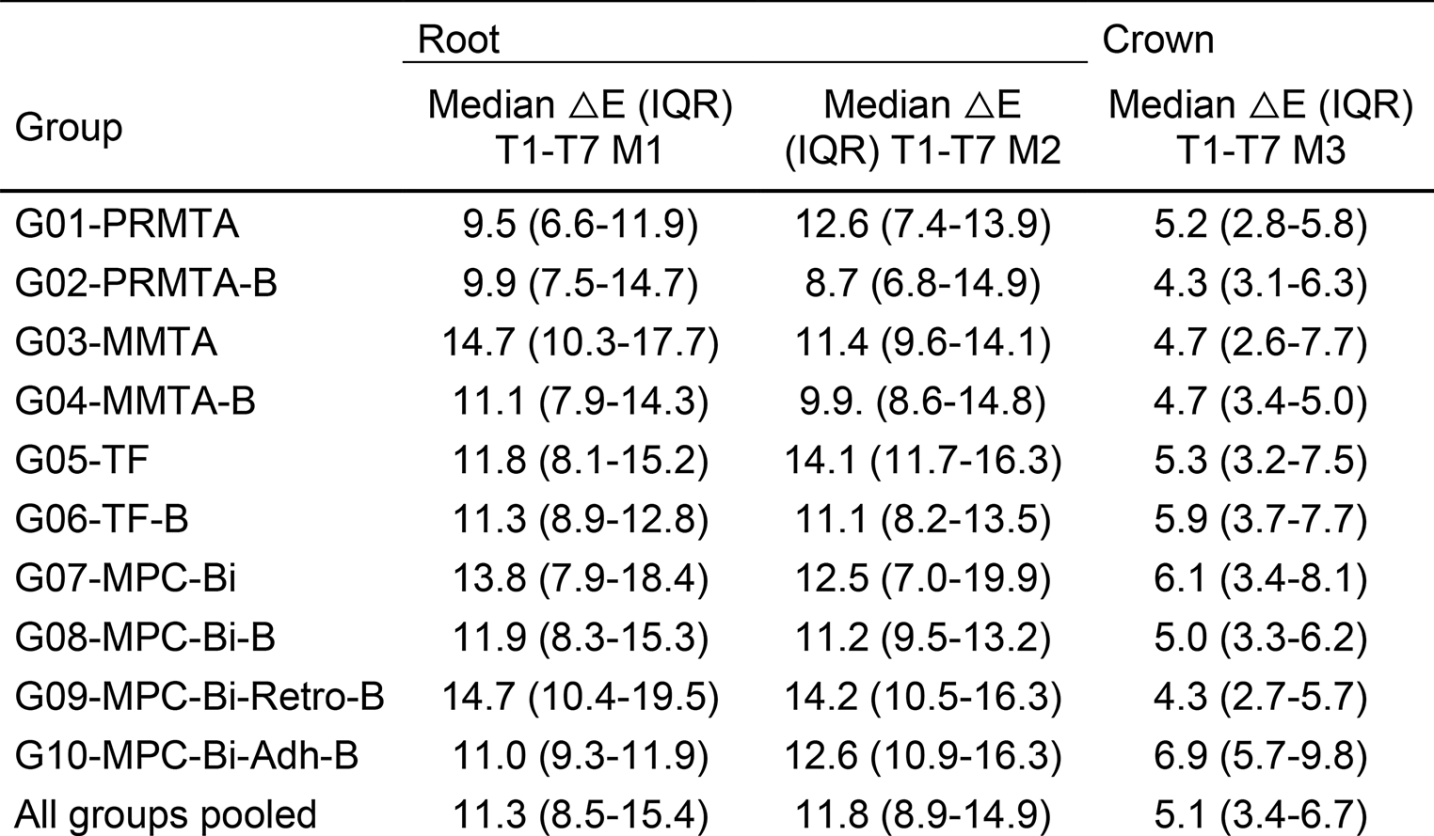
Groups of specific test materials and mixing ratios used for apical plug treatment (APT) with proroot MTA (PRMTA), Medcem MTA (MMTA) or totalfill BC RRM fast set putty (TF) with and without bovine blood (B), and for retrograde and orthograde APT with Medcem medical Portland cement (MPC) plus Bi_2_O_3_ (Bi) with and without bovine blood (B) after adhesive sealing (Adh) of the coronal dentin using scotchbond universal adhesive (3 M, Neuss, Germany) as reported previously [22]

The cements were mixed according to the specifications in Table 1. In groups 7 to 10, Bi_2_O_3_ was added to Medcem Medical Portland Cement to assess its influence on tooth color stability. During the obturation procedure, each specimen was securely positioned in silicone sockets (Silaplast, Detax, Ettlingen, Germany). Apical plugs of each HCSC were placed from the apical terminal point using an applicator (LyDenti, Berlin, Germany) with a 1.2 mm tip and a dental microscope (Zeiss, Jena, Germany) with 0.6 × magnification. The material was condensed with pluggers and paper points to obtain a thickness of 4 mm. All HCSCs were placed using an orthograde approach, except for group 9. Special precautions were taken to ensure the coronal cavity remained uncontaminated during the orthograde placement. After HCSC placement, all dentinal walls were cleaned using microbrushes soaked in distilled water. The remaining portion of the root canal was obturated with sealer (AH Plus, Dentsply DeTrey, Konstanz, Germany) and warm gutta-percha, placed up to 1 mm below the cementoenamel junction (CEJ) using a warm vertical compaction technique. The access cavity was subsequently cleaned with cotton pellets moistened with 70% ethanol.

In group 9, using the same vertical compaction technique as in the other groups, the entire root canal, up to 1 mm below the CEJ, was initially filled with sealer and gutta-percha. Subsequently, 4 mm of the apical portion of the root canal filling was removed via the apical foramen using heat pluggers, and the space was then filled with a mixture of Medcem Medical Portland Cement, Bi_2_O_3_, and bovine blood.

In group 10, the dentin was adhesively sealed within the access cavity down to 1 mm below the CEJ using a universal adhesive (ScotchBond Universal Adhesive, 3 M, Neuss, Germany) before APT. This seal was subsequently removed with a diamond bur after the apical plug and the remaining part of the root canal filling had set.

Across all groups, the coronal cavities were restored with a flowable resin-based composite (3 increments of SDR, Dentsply DeTrey, Konstanz, Germany) following etching of the enamel with 37% phosphoric acid (Omni-Etch, Omnident, Rodgau, Germany) and the application of a universal adhesive (ScotchBond Universal Adhesive, 3 M, Neuss, Germany). Light curing of the adhesive and the increments of resin-based composite was performed for 20 s using an LED curing light (EliparTM Free Light 2, 3 M, Seefeld, Germany). Each tooth was then stored individually in a labeled tube containing distilled water at a temperature of 37 °C in a darkroom.

### Spectrophotometric color determination

**Fig. 1:**
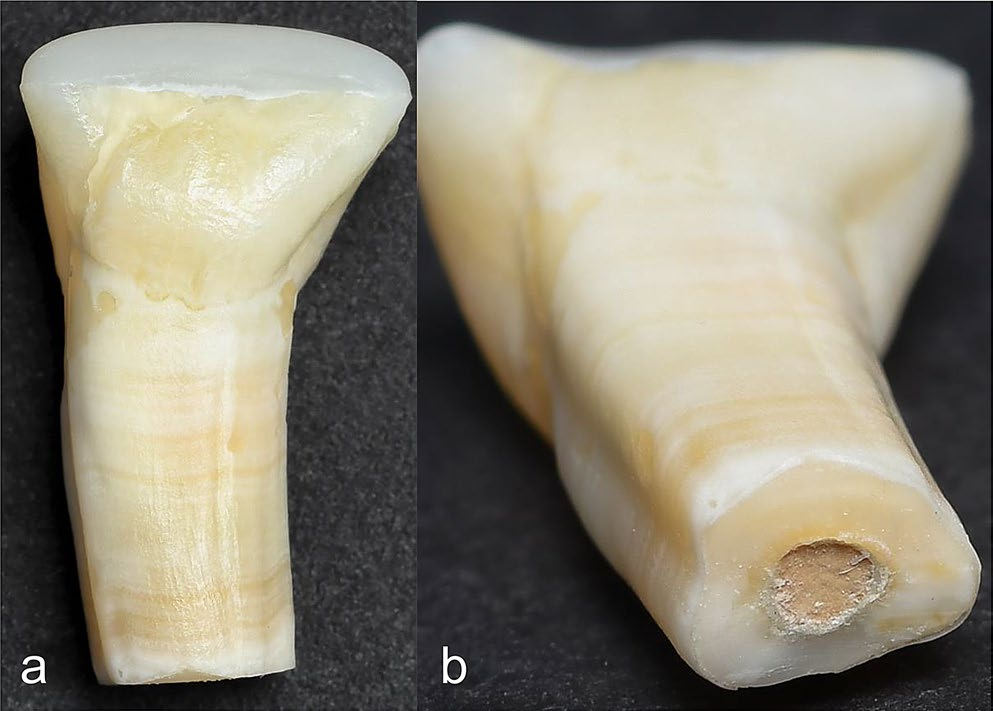
Representative specimens (HCSC plug admixed with blood) displaying visible discoloration on the apical and central root surfaces after 72 months following APT (**a**: front view, **b**: apex view)

Color measurements were performed under standardized illumination conditions using a calibrated spectrophotometer (VITA Easyshade V, VITA Zahnfabrik, Bad Säckingen, Germany), following the L*a*b system (Commission Internationale de l’Eclairage) as reported previously [22]. The standardization of the measurements included consistent light conditions (10 W, 50/60 Hz, 46 mA) in a dark room with the stream of light from the same angle without any reflections. Each measurement started with a consistent process of a white balance procedure calibrating the spectrophotometer followed by drying the sample and measuring three times on each measuring surface. Tooth color was measured on three different measuring surfaces (M1 = middle of the cement plug, M2 = middle of the root filled with gutta-percha, M3 = middle of the tooth crown). The values of change in color (ΔE) represented the difference between two color readings. Measurements were taken 72 months (T7) after the filling placement (see Fig. 1a-b), with each reading repeated three times and averaged. The study utilized color measurement data recorded before root canal filling (T0), 24 h post-treatment (T1 = baseline), and at one (T2), three (T3), six (T4), twelve (T5), twenty-four months (T6) [22], and 72 months (T7) after obturation. During the follow-up period the samples were stored at 37 °C in a heating cabinet (Memmert) throughout the aging process. The distilled water was changed each month and the storage was supervised from the same operator over the entire 72-month period. A researcher (F.S.) performed spectrophotometric investigation and analysis.

**Fig. 2:**
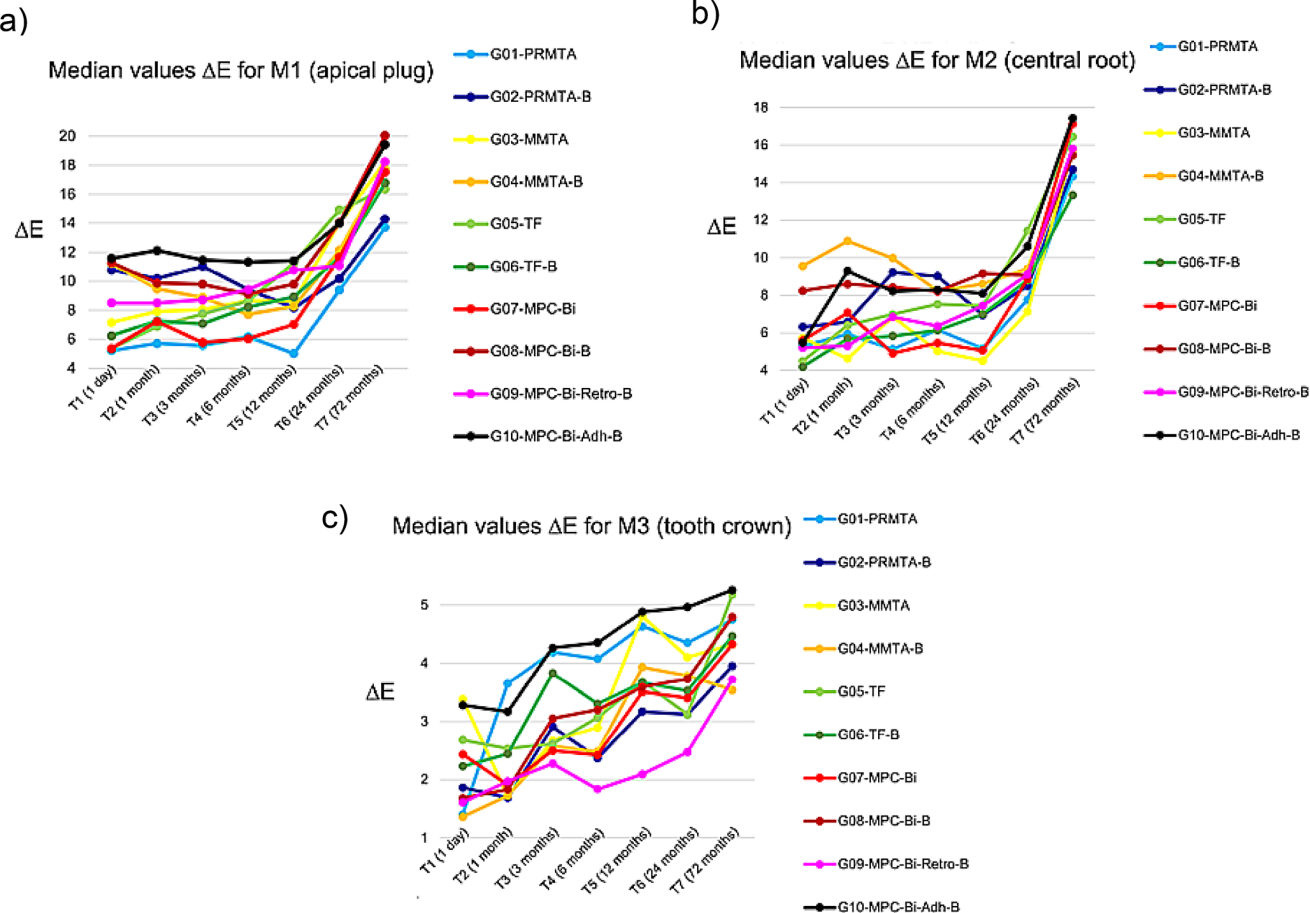
Median color change ∆E of each group (**a**: on the apical plug surface (M1); **b**: on the gutta-percha/sealer surface (M2); **c**: on the tooth crown/resin composite surface (M3)) in relation to T0 (baseline control: color measurement data recorded before root canal filling)

For each group, ∆E values were calculated as the color difference between T1 (24 h) and T7 (72 months), as determined by spectrophotometry (Table 2, see Fig. 2a-c). The degradation effect of the materials inside the root canal was assessed independently of the material’s inherent color by comparing the color differences between the empty root canal before treatment (T0; baseline control) and the filled root canal 24 h after filling (T1). Color difference (∆E*_ab_), here also denoted as ∆E, was calculated as a function of the change in lightness (∆L) with L* for the lightness from black (0) to white (100), a* from green (−) to red (+), and b* from blue (−) to yellow (+), as described previously [22].

### Statistical analysis

Medians and interquartile ranges of ∆E (∆E*_ab_) were calculated for each group at the specified time points. Statistical analyses included one-way analysis of variance (ANOVA) with post-hoc tests, the Friedman test, Mann-Whitney U-test, t-test, and post-hoc tests with Bonferroni correction. An unblinded statistician conducted these analyses using IBM SPSS Statistics software (Version 24, IBM, Endicott, USA). The normal distribution of the data, a prerequisite for conducting ANOVA, was assessed using the Shapiro-Wilk test. The level of significance was set at α = 0.05.

## Results

After 72 months, ∆E increased substantially and color differences exceeded the thresholds of human perception on all measuring surfaces (M1-3). Color differences on all measuring surfaces were statistically significant at 72 months (T7) compared with other time points (T1, T6), as determined by pairwise post-hoc-tests of the Friedman-test (*p* <.001; effect size: 0.252 (T6-T7) < *r* <.668 (T1-T7)). The color difference was the highest on the apical plug surface (M1) and the lowest on the tooth crown surface (M3) (Table 2). ANOVA-tests revealed that there was no significant difference in ∆E on M1, M2, or M3 between the groups (*p* >.05).

**Table 2:** Median color change ∆E with interquartile range (IQR) from 24 h (T1) to 72 months post-treatment (T7) on the apical plug (M1), gutta-percha/sealer (M2), and tooth crown/resin composite surfaces (M3) of all groups in relation to T1

Group	Root		Crown
	Median △E (IQR) T1-T7 M1	Median △E (IQR) T1-T7 M2	Median △E (IQR) T1-T7 M3
G01-PRMTA	9.5 (6.6–11.9)	12.6 (7.4–13.9)	5.2 (2.8–5.8)
G02-PRMTA-B	9.9 (7.5–14.7)	8.7 (6.8–14.9)	4.3 (3.1–6.3)
G03-MMTA	14.7 (10.3–17.7)	11.4 (9.6–14.1)	4.7 (2.6–7.7)
G04-MMTA-B	11.1 (7.9–14.3)	9.9. (8.6–14.8)	4.7 (3.4-5.0)
G05-TF	11.8 (8.1–15.2)	14.1 (11.7–16.3)	5.3 (3.2–7.5)
G06-TF-B	11.3 (8.9–12.8)	11.1 (8.2–13.5)	5.9 (3.7–7.7)
G07-MPC-Bi	13.8 (7.9–18.4)	12.5 (7.0-19.9)	6.1 (3.4–8.1)
G08-MPC-Bi-B	11.9 (8.3–15.3)	11.2 (9.5–13.2)	5.0 (3.3–6.2)
G09-MPC-Bi-Retro-B	14.7 (10.4–19.5)	14.2 (10.5–16.3)	4.3 (2.7–5.7)
G10-MPC-Bi-Adh-B	11.0 (9.3–11.9)	12.6 (10.9–16.3)	6.9 (5.7–9.8)
All groups pooled	11.3 (8.5–15.4)	11.8 (8.9–14.9)	5.1 (3.4–6.7)

There were no significant differences in ∆E on M1, M2, or M3 between groups with and without admixture of blood at T7 (Shapiro-Wilk-test, *p* >.05). Groups with admixture of blood appeared visually darker than those without blood at T7. The analysis of the raw data was performed in order to detect differences in terms of lightness [L]. In the PRMTA groups (G01 with median L 88.4 (85.2–89.8) vs. G02 with median L 82.5 (80.3–84.2)), L values were significantly lower with admixture of blood on M1 (Mann-Whitney-U-test, *p* =.012), which confirmed specifically the visual impression.

There were no significant differences in ∆E on M1, M2, or M3 between groups with and without admixture of Bi_2_O_3_ at T7 (Shapiro-Wilk-test, *p* >.05).

A retrograde placement (G09-MPC-Bi-Retro-B) showed a tendency toward more discoloration of all measuring surface (M1, M2, and M3) after 72 months (T7) than orthograde placement (G08-MPC-Bi-B), but the differences were very small and not statistically significant (t-test, *p* >.05).

No effect of prior adhesive dentin sealing of the coronal cavity (G10-MPC-Bi-Adh-B vs. G08-MPC-Bi-B; t-test, *p* >.05) could be detected after 72 months (T7).

Overall, the coronal discolorations (M3) were very mild compared with the substantial discolorations of the root (M1 and M2) after six years. They were often above the human perception threshold.

## Discussion

This in-vitro study continued the spectrophotometric color determination of bovine teeth, assessing tooth discoloration in the aesthetic relevant zone induced by HCSC-based apical plugs with and without blood contamination, up to 72 months. Apical plugs of the tested HCSCs did not cause discoloration of bovine tooth crowns after a six-year follow-up under various simulated clinical conditions, provided they were carefully placed, avoiding direct contact with the coronal dentin. However, a substantial color change was observed on the apical and central parts of the root from the 24- to the 72-month period. The extent of discoloration on all measuring surfaces did not differ between the HCSC test groups after six years. There were no significant effects from blood contamination during the HCSC setting time, the type of HCSC placement (ortho-/retrograde), or the adhesive sealing of the dentin in the pulp chamber in terms of tooth discoloration after six years.

There was no relevant change in tooth color after 12 months [22]. However, tooth color increased significantly at the apex, where the apical plug was placed, after 24 and 72 months. The tooth crown did not show any HCSC-related discoloration after the six-year observation period. Delayed discoloration was expected due to the use of bovine teeth of 4 to 6-year old donor animals, which had a high number of sclerotic dentinal tubules [22]. Thus, stronger discoloration occurred after the observation period of a few years, but it was limited to the apical and central parts of the root. It is suggested that the thickness of the dentinal wall affects tooth discoloration. Yassen et al. reported inconsistencies between the effects of endodontic materials in bovine teeth and human teeth [33], mainly attributed to differences in dentin permeability [34]. Human teeth with open apices usually have thin dentinal walls and wide dentinal tubules allowing penetration of chemical products that cause discoloration. If such teeth require root-end closure with HCSC plugs, clinically relevant tooth discolorations will occur more quickly in human than in bovine teeth. However, the 6-year results of the present study revealed a slow increase in tooth discoloration within bovine dentin areas close to the placed plug.

There is only one in-vitro study with a similar mid-term investigation evaluating tooth color changes of products from calcium-silicate based root canal sealers in the entire human root up to three years [35]. This study revealed acceptable levels of discoloration with a maximum at the 6-month follow-up in incisal and cervical portions. There was no relevant increase in color change during the following observation period. In agreement with the results of our study, clinical efforts must focus on avoiding contamination of coronal dentin with HCSC material during plug placement or root filling, and on thoroughly cleaning the pulp chamber after obturation.

Potential discoloring interactions between HCSCs, sodium hypochlorite and Bi_2_O_3_ were minimized by using the previously reported irrigation protocol (sodium hypochlorite and ethylenediaminetetraacetic acid) for cleaning the dentinal walls [22, 36]. Additionally, the specimens were stored in distilled water in an incubator at 37° C between each measurement phase. The current recommendations for tooth storage were generally followed, except for the omission of 0.10% thymol for keeping teeth moist in water and the use of chloramines for a short period after tooth extraction [34].

All four tested HCSCs caused ongoing tooth discoloration in the apical and central areas of the root from the 24-month to the 72-month observation period. There were no statistically significant differences between the materials.

Increased tooth staining may be caused by the admixture of bovine blood during the setting time of HCSCs, which is a clinically relevant condition [12, 13]. Blood components can be absorbed by endodontic cements, and subsequent interactions between light and the physiological degradation of erythrocytes may occur [21]. The strongest color changes occurred in teeth with the retrograde placement of the Bi_2_O_3_ containing MPC with blood (G09-MPC-Bi-Retro) after 72 months (T7) (M1: median ΔE = 14.7; M2: median ΔE = 14.2). Orthograde-placed HCSC materials with blood showed a very similar or milder discoloration of the root compared with their corresponding groups without blood. This effect seemed to increase from the apical part (M1) to the central part (M2) of the root. Furthermore, this effect was consistent for Bi_2_O_3_ containing groups (G01-PRMTA, G07-MPC-Bi) and groups without Bi_2_O_3_ (G02-MMTA, G05-TF). These findings might be explained by a decrease in lightness (ΔL-values) of these specimens resulting in a darker appearance.

Within the limitations of the present study setup no long-term discolorations related to Bi_2_O_3_ could be detected. This is consistent with the short-term results reported by Dettwiler et al., which showed that Bi_2_O_3_ has limited potential for inducing tooth discolorations [36]. Moreover, the 72-month data indicated that blood does not have a provable impact on long-term tooth discoloration at all, which contrasts with our reported results after 24 months [22]. The measured lightness values showed a tendency for the groups with admixed blood to appear darker; however, there was no consistent correlation in the relevant groups with and without the admixture of blood on the measuring surfaces on the root or the tooth crown.

In agreement with our previously reported data, no long-term benefit of prior dentin sealing on coronal discoloration related to apical plugs could be detected, which contrasts with findings of another group [37]. This discrepancy can be explained by the different study setups. As mentioned above, plug placement can be performed more easily in bovine teeth owing to both the larger access cavities and greater diameter of the root canal. Contamination of the coronal dentin walls may occur more frequently during clinical treatment in human teeth than in the animal model [22].

Further studies must consider the evaluation of color changes by using the modern CIEDE2000 system, which is reported to provide more refined results and detect subtler color differences [38, 39], compared with the CIE Lab* formula. Additionally, the CIEDE2000 system is able to offer superior adjustments in assessing small visual tolerances, as found in dental clinics [40], and in terms of the present study’s issue focusing on the clinical relevance of coronal discoloration.

Within the 72-month observation period of the present study, no HCSC-related discoloration was observed in the aesthetic zone. A review concluded that the staining potential of HCSCs varies and is often below or close to the threshold of human perception, with a ∆E value of 3.3 [10]. In the present study, the median color increase for each HCSC plug did not exceed the value of ∆E 2.0 on the tooth crown from the 24-month to the 72-month observation period. It remains unclear how long it will take before clinically relevant color changes can be detected with adequate HSCS plug placement.

## Conclusions

Apical plugs of the tested HCSCs did not cause discoloration of bovine tooth crowns after a six-year follow-up, provided they were carefully placed to avoid direct contact with coronal dentin. However, significant root discoloration was observed in this long-term investigation. In general, root canal fillings of gutta-percha and sealer must be placed adequately below the CEJ followed by an adhesive restoration of the access cavity.

## Data Availability

No datasets were generated or analysed during the current study.
